# Leadless pacemaker implantation via right jugular approach in a patient with a bicaval valve system: a case report

**DOI:** 10.1093/ehjcr/ytag291

**Published:** 2026-04-27

**Authors:** Krister Kuhnhardt, Huong Lan Phan, Ferdinand Witt, Holger Nef, Stephan Fichtlscherer

**Affiliations:** Heart and Vascular Center Segeberger Kliniken, Am Kurpark 1, Bad Segeberg 23795, Germany; Heart and Vascular Center Segeberger Kliniken, Am Kurpark 1, Bad Segeberg 23795, Germany; Heart and Vascular Center Segeberger Kliniken, Am Kurpark 1, Bad Segeberg 23795, Germany; Heart and Vascular Center Segeberger Kliniken, Am Kurpark 1, Bad Segeberg 23795, Germany; Heart and Vascular Center Segeberger Kliniken, Am Kurpark 1, Bad Segeberg 23795, Germany

**Keywords:** Tricuspid regurgitation, Leadless pacemaker, Aveir™, TricValve®, Jugular access, Advanced heart failure, Case report

## Abstract

**Background:**

Tricuspid regurgitation in elderly patients with multiple comorbidities presents unique challenges, especially after prior valve interventions and when advanced heart failure symptoms persist. New device-based therapies such as bicaval valve stents (TricValve®) can offer alternatives for patients not suitable for surgery but may complicate pacemaker implantation.

**Case summary:**

We report an 85-year-old woman with a history of coronary artery disease, prior valve-in-valve transcatheter aortic valve implantation (TAVI) for a degenerated aortic prosthesis, and permanent atrial fibrillation. She presented with decompensated right heart failure due to severe secondary tricuspid regurgitation, despite optimal medical treatment. After the heart team discussion, surgical repair and transcatheter tricuspid valve replacement were denied for prohibitive risk. A TricValve® system was successfully implanted to treat symptomatic caval reflux.

Several days later, she developed complete atrioventricular block with recurrent syncope. A leadless pacemaker (Aveir VR™) was implanted via the right internal jugular vein and navigated safely through the superior cava prosthesis. Device deployment was successful, with no acute procedural complications; however, the patient died shortly thereafter due to pneumonia.

**Discussion:**

This case demonstrates the feasibility of leadless pacemaker implantation in the presence of a bicaval valve system and complex, high-risk anatomy. While transvenous pacemaker implantation may still be feasible with a superior vena cava valve prosthesis in place, it can cause unwanted interference, potentially impairing valvular function and increasing the risk of endocarditis compared with leadless pacemaker solutions. However, the findings are limited to acute procedural success.

Learning points

- Transcatheter tricuspid interventions are increasingly performed in elderly, high-risk patients and may substantially complicate pacemaker implantation.
- Leadless pacemaker systems offer an important alternative in this setting, as they avoid transvalvular leads and can provide safe and effective pacing even in the presence of complex prosthetic valves.
- Implantation of an AVEIR VR leadless pacemaker through a bicaval TricValve® system via the right internal jugular vein is technically feasible.

## Introduction

Tricuspid regurgitation is particularly common in elderly and high-risk patients; however, guideline-directed surgical therapy is often not feasible due to frailty and high procedural risk.^1^ Novel transcatheter approaches, including bicaval valve stents such as the TricValve®, provide symptomatic relief even in patients deemed not suitable for conventional procedures.^2^ The TricValve® Bicaval Valve System consists of two self-expanding bioprosthetic valves designed to reduce caval reflux in severe tricuspid regurgitation without interacting with the native tricuspid valve.^3^

However, device-related complications—especially the need for permanent pacing—may arise after these procedures.^4^ Therefore, leadless pacing has emerged as an attractive solution to minimize further bioprosthetic valve interference.^5^

## Summary figure

**Figure:**
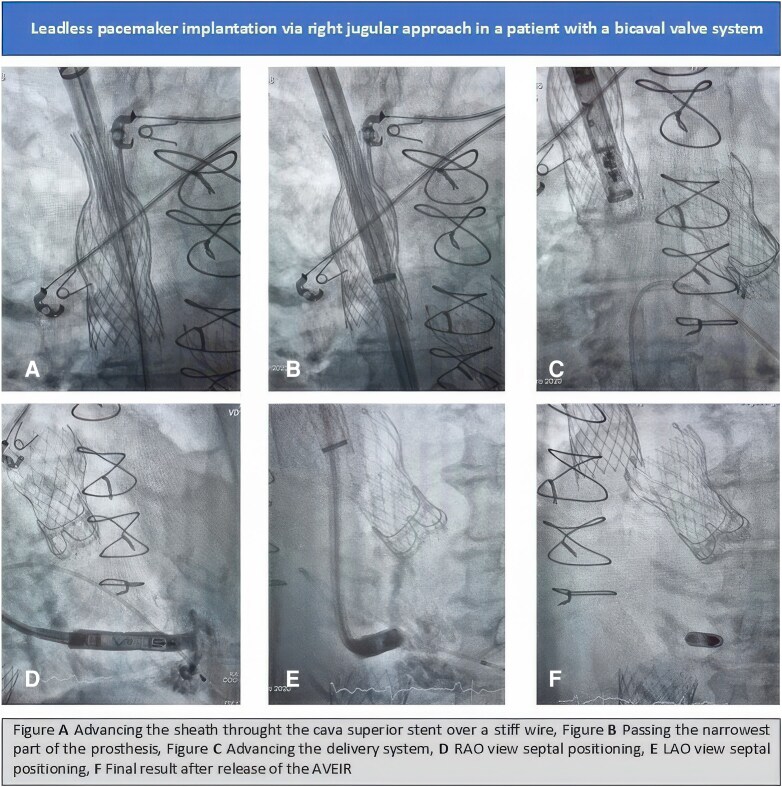


## Case presentation

**Figure 1:**
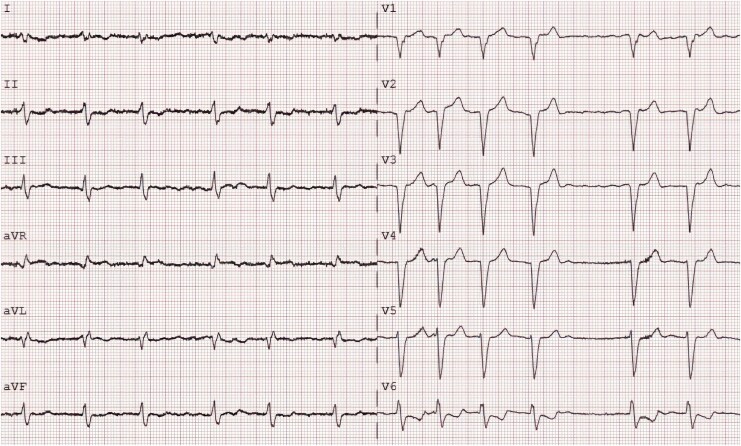
In total, a 12-lead ECG prior to TricValve® implantation, 25 mm/s, showing left bundle branch block and atrial fibrillation, as well as marked right axis deviation.

An 85-year-old woman with coronary artery disease and recent valve-in-valve TAVI for a degenerated aortic bioprosthesis developed persistent left bundle branch block post-procedure, shown in *Figure 1*. Despite optimal medical therapy, she experienced recurrent decompensations due to severe secondary tricuspid regurgitation.

**Figure 2:**
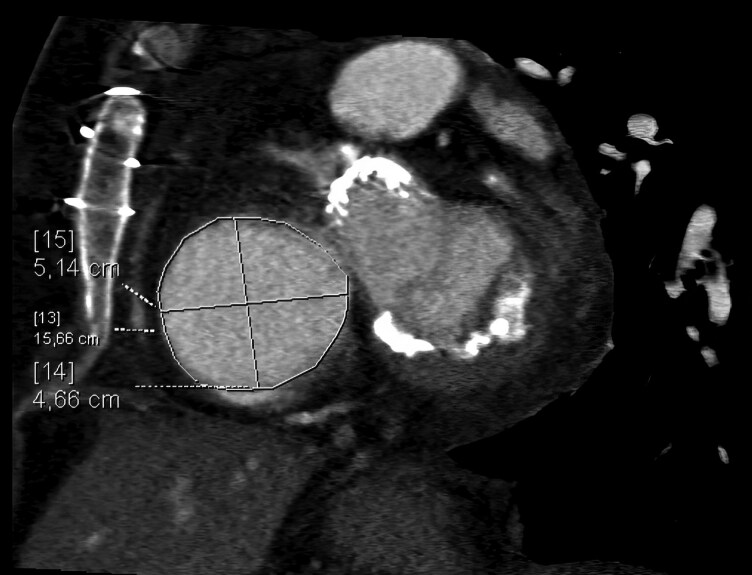
CT assessment of the tricuspid valve annulus with corresponding measurements.

Given her age, frailty, and comorbidities (TRISCORE 7, 34% predicted in-hospital mortality), surgical tricuspid valve replacement was denied. Transcatheter tricuspid edge-to-edge repair (T-TEER) was considered unsuitable because of a massively dilated annulus (51 mm) shown in *Figure 2*, severe leaflet malcoaptation, and markedly dilated right atrium. Transcatheter tricuspid valve replacement (TTVR) was considered invasive, given the patient’s frailty. After a heart team discussion, a TricValve® bicaval stent system was implanted to manage symptomatic tricuspid regurgitation in this high-risk patient.

**Figure 3:**
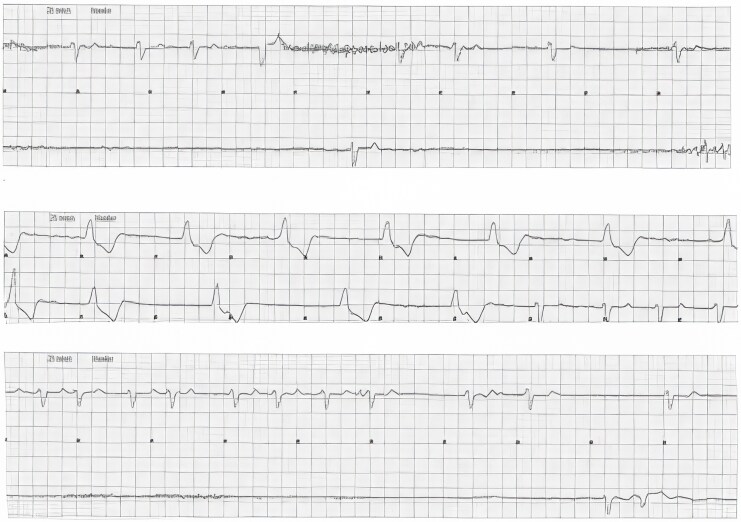
Telemetry ECG (25 mm/s) recorded during syncopal episodes, showing relevant episodes of asystole and a change in QRS vector with a regular escape rhythm, suggestive of complete atrioventricular block.

Four days after implantation, she developed recurrent episodes of complete AV block with syncope, shown in *Figure 3*. A temporary pacemaker was inserted via the right femoral vein, but catheter manipulation was challenging due to the inferior TricValve® stent. Coronary sinus lead placement was excluded because the superior vena cava prosthesis would impede catheter manoeuvrability and risk compromising prosthesis stability. Conventional transvenous pacing through the prosthesis was also considered unsafe, and an epicardial lead was not considered due to the high operative risk.

Given her urgent and persistent need for pacing, a leadless pacemaker (Aveir VR™) was chosen, also considering its active fixation and full retrievability, allowing potential repositioning in this complex anatomical setting. To avoid complications from femoral access (already used for the temporary pacer) and a potentially crucial over 90° angulation for the delivery system in the valve prosthesis in the inferior vena cava, the right internal jugular vein was chosen.

**Figure 4:**
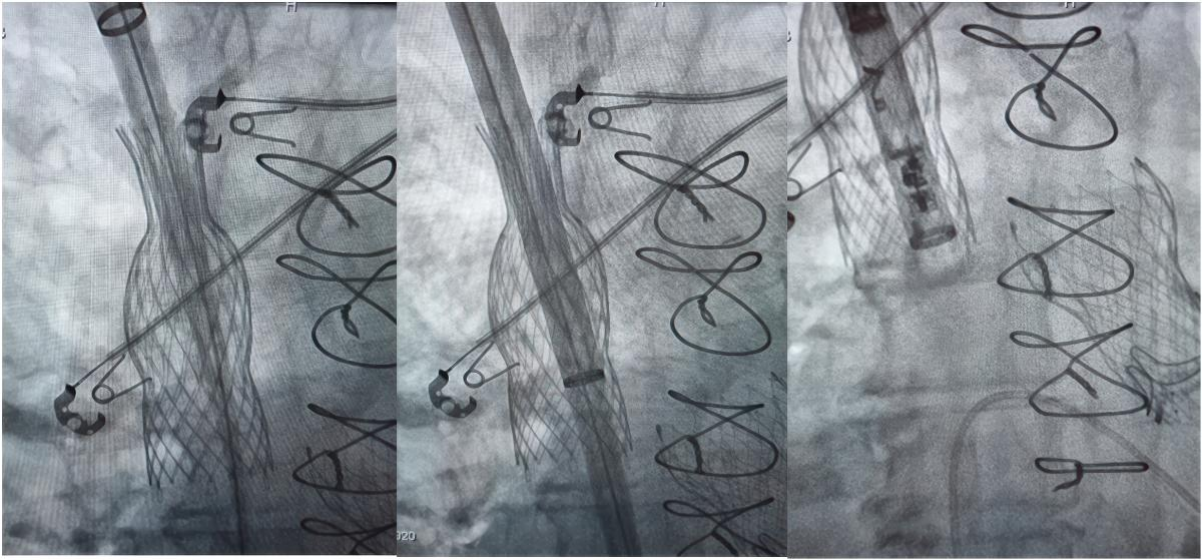
The delivery sheath was advanced through the superior vena cava prosthesis over a stiff guidewire and carefully positioned just at the distal end of the prosthesis to minimize mechanical manipulation of the valve.

Under ultrasound guidance the vein was safely punctured and after predilation the 25F, 50 cm sheath was advanced through the superior vena cava TricValve® stent into the right atrium. Despite the stent’s narrow upper diameter (20 mm), careful fluoroscopic control enabled atraumatic passage of the delivery system over a stiff guidewire (Amplatz Super Stiff^TM^, Boston Scientific) through the prosthesis and across the native tricuspid valve. The delivery sheath was carefully advanced just to the distal end of the prosthesis, minimizing mechanical manipulation of the valve (*Figure 4*). We considered this step, along with the retrieval of the sheath from the valve, to be the most challenging part of the procedure.

Sheath advancement and retrieval were performed under continuous fluoroscopic guidance with careful coaxial alignment to minimize the risk of leaflet interference or prosthesis distortion. The Aveir™ pacemaker was successfully fixed onto the right ventricular septum, with excellent acute electrical parameters (sensing: 6.7 mV, pacing threshold: 0.75/0.4 V/ms, impedance: 630 ohm). Post-procedural angiography confirmed preserved valve integrity. No procedural complications occurred. Postinterventional hemostasis at the puncture site in the right internal jugular vein was achieved using a ‘figure-of-eight’ suture. Total procedure time was 35 min with a dose-area product of 970 cGy·cm^2^.

Device interrogation on the first postoperative day demonstrated excellent electrical parameters (sensing 7.4 mV, pacing threshold 0.5 V/0.4 ms, impedance 560 ohm) with a pacing burden of 11%. Unfortunately, the patient died three days after the procedure due to severe pneumonia with respiratory failure. In accordance with her expressed wishes, no invasive life-sustaining measures were initiated. The patient's death was unrelated to the pacemaker implantation.

## Discussion

This case highlights the increasingly complex scenarios for device therapy after percutaneous treatment of tricuspid regurgitation, especially with novel prosthetic options like the TricValve®. AV block requiring pacing is a recognized complication following transcatheter valve therapy, with recent incidences up to 11%.^1,6^

Atrioventricular block following TricValve® implantation has been described.^7^ However, because the TricValve® system is implanted in a heterotopic position in the superior and inferior vena cava and does not exert direct interaction with the atrioventricular node or His-Purkinje system, a direct causal relationship between the prosthesis itself and AV block remains uncertain. In our case, the previously implanted TAVI, performed four months earlier, may have also contributed as a potential risk factor for the development of a ‘late’ new atrioventricular block.

Conventional transvenous pacing after tricuspid procedures carries the risk of worsening regurgitation, interference with prosthetic structures, or technical failure.^1,8–10^ Leadless pacemakers avoid crossing the tricuspid valve and therefore reduce mechanical stress and lead-related infectious complications.^2,4,5,8,10^ Recent consensus statements now recommend individualized heart team decision-making for elderly, multimorbid patients with tricuspid regurgitation and device needs.^1,2,6^ Surgical and epicardial solutions, as well as coronary sinus lead placement, may be impossible if prosthesis placement narrows conventional venous access or alters anatomy.^1,2,4,6,10^

Jugular vein access for leadless systems is documented as safe and effective in patients with challenging anatomy or bioprosthetic valves—several cases and small series have described successful navigation of the superior vena cava stents, valve rings, and even other prosthetic devices by using careful fluoroscopy and individualized access selection.^4,5,10,11^ This approach involves passing a large introducer sheath into the right atrium, which can be especially challenging if the prosthesis is narrow or tortuous; careful imaging and planning are critical for a safe outcome.^4,5,11,12^ Successful leadless pacemaker implantation via femoral access has been previously reported in a patient with a bicaval valve system, supporting the feasibility of this approach.^13^ Nevertheless, the use of a 25F delivery sheath within a 20-mm SVC prosthesis carries inherent risks, including leaflet interference, prosthesis distortion or displacement during sheath advancement or retrieval, which were mitigated by strict fluoroscopic guidance and careful coaxial alignment. Postprocedural assessment of vena cava prosthesis function should be performed by fluoroscopy and angiography, confirming preserved leaflet mobility and valve integrity.

Leadless pacemaker implantation via the right internal jugular vein has been demonstrated to be feasible and safe in a consecutive series of patients, suggesting that alternative vascular access routes may expand the applicability of leadless systems.^14,15^ Leadless pacing following tricuspid valve interventions without the presence of a bicaval valve system has been previously described.^4,12^

While the leadless pacemaker implantation was technically successful with excellent acute device performance, the patient ultimately died from respiratory failure due to a pulmonary infection. This underlines the overall clinical complexity and vulnerability of this fragile patient population.

To the best of our knowledge, this is the first reported case describing successful Aveir VR™ leadless pacemaker implantation via the right internal jugular vein through a TricValve® bicaval prosthetic system. This case underscores how modern device options, careful procedural imaging, and collaborative decision-making provide solutions in unprecedented clinical scenarios, offering pacing when conventional techniques are contraindicated or limited.^1,2,4,6,10^ However, the findings are limited to acute procedural success, as the patient died shortly thereafter from pneumonia.

## Lead author biography


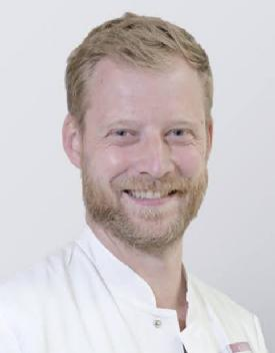
Senior Physician, Head of Device Therapy, Heart Center, Segeberger Kliniken

**Consent:** The authors confirm that the patient provided written informed consent for the publication, in accordance with COPE guidelines.

## Data Availability

The data underlying this article will be shared on reasonable request to the corresponding author.
